# Relations between vitamin D3, total and specific IgE for house dust mites in atopic dermatitis patients

**DOI:** 10.1038/s41598-020-77968-1

**Published:** 2020-12-02

**Authors:** Hassan M. Ibrahim, Moustafa A. El-Taieb, Mohammed H. Hassan, Abd Allah E. Mohamed, Ebtihal A. Kotop, Osama H. Abd-ellah, Eisa M. Hegazy

**Affiliations:** 1 Dermatology, Andrology and Venereology, Qena Faculty of Medicine, South Valley University, Qena, 83523 Egypt; 2 Department of Dermatology, Andrology and Venereology, Aswan Faculty of Medicine, Aswan University, Tingar, Egypt; 3 Department of Medical Biochemistry, Qena Faculty of Medicine, South Valley University, Qena, Egypt; 4 Clinical Pathology, Qena Faculty of Medicine, South Valley University, Qena, Egypt; 5 Dermatology, Venereology and Andrology, Qena University Hospital, South Valley University, Qena, Egypt; 6 Parasitology Department, Qena Faculty of Medicine, South Valley University, Qena, Egypt; 7 Department of Dermatology, Andrology and Venereology, Qena Faculty of Medicine, South Valley University, Qena, Egypt

**Keywords:** Biochemistry, Immunology, Medical research

## Abstract

Atopic dermatitis (AD) is a chronic recurrent inflammatory skin disease. There are a lot of evidences on the importance of vitamin D and house dust mite (HDM) allergens in the etiology and course of AD. The objectives of this study are to evaluate the relation between vitamin D3 level and house dust mites (HDM) Dermatophagoidspecies sensitization in pathogenesis of atopic dermatitis. Cross-sectional design study was conducted on 50 atopic dermatitis patients. Blood analysis were done to determine level of vitamin D3, total IgE by fluorescent immunoassay & specific IgE for HDM (d1, d2) & other inhalant allergens by ELISA test. There was significant high negative correlation with the specific IgE for HDM (r =  −0. 62, p < 0.001) and vitamin D3. & there was non-significant minimal negative correlation with the specific IgE and other inhalant allergens (r = − 0.10, p > 0.05). There was a statistically significant relation between level of vitamin D3 and atopic dermatitis severity and sensitization to HDM and other allergens.

## Introduction

Atopic Dermatitis (AD) is a chronic inflammatory skin disease characterized by episodes of severe itching, dry skin and erythematous lesions. AD generally presents as an episodic disease with repeated flare-ups; however, it may also be continuous. AD improves or resolves in adulthood in most of patients^1,2^.

AD has a prevalence of up to 30% in children and up to 3% in adults, although it can present at any age, up to 85% of patients are symptomatic before 5 years of age. There is an increase in the prevalence of adult-onset disease nowadays^3^. There is no accurate data about the prevalence of AD dermatitis in Qena governorate, but in their study, Abdel- Hafez et ali., found that AD prevalence of 1% in rural areas of Assiut governorate which is located nearby Qena and has the nearly the same climate, environmental and social circumstances^4^.

The pathogenesis of AD is still not fully understood. AD is now mostly recognized as a multifactorial disease that develops due to interaction of multiple factors such as susceptibility genes, environmental factors, impaired skin barrier integrity, and immune dysregulation^5^. Recent genome-wide association studies identified loci correlated with autoimmune regulation, including genes associated with regulation of innate host defenses and T-cell function^6^.

In patients with genetic defects in certain genes such as filaggrin gene, environmental stimulations as detergents and soaps lead to decrease in hydration and extracellular lipids in the stratum corneum with subsequent production of antigens and enhancement of inflammation^7^.

Antimicrobial peptides (AMPs) such as cathelicidin and defensins have an important role in skin barrier function and immune signaling. Previous studies reported that vitamin D has an important role in expression of cathelicidin and defensins in the skin through its action on the Toll-like receptor 2^8^. Also it is well known that vitamin D has a positive role on adaptive immune responses such as maturation and activation of dendritic cells and T-cell^9^.

Patients with AD has defect in innate and cell mediated immunity which also was correlated with vitamin D^8,10^. Vitamin D deficiency can negatively affect skin barrier function and enhance the triggering of AD^11^. It was also suggested that vitamin D deficiency may increase the susceptibility of skin of patients with AD to be superinfected by Staphylococcus aureus^12^.

The role of airborne allergens in AD has been studied with conflicting results. Ig E sensitization to HDM was detected in adult patients with AD suggesting a role in AD pathogenesis ^13^. HDM itself may trigger atopic flares in patients with AD even without elevated Ig E through non allergic mechanisms^14,15^. In an in-vitro study, it was found that HDM stimulate the expression of IL-22Ra and CCL17/thymus and activation-regulated chemokine (TARC) in the keratinocyte line. It also enhances the increase secretion of IL-22 by T cells^16^.

In the present study we investigated Vitamin D3, Total and specific IgE for HDM & its relations to the pathogenesis and the course of AD.

## Patients and methods

### Patients

This cross-sectional observational study was conducted on 50 patients with AD recruited from the outpatient clinic of Dermatology, Qena Faculty of Medicine, South Valley University, Egypt in the period between May 2018 and May 2019. According to Rajka and Langeland score^17^, patients were classified into three groups, either the mild group (3–4) or moderate group (4.5–7.5) or the severe group (8–9). Age ranged age ranged from 2 month to 16 years.

Patients with asthma, rhinitis, other respiratory diseases, scabies, parasitic infestations, pityriasis rubra pilaris, allergic and contact dermatitis, seborrhoeic eczema have excluded from the present study.

## Methods

### Blood samples and laboratory investigations

5 ml of venous blood were drawn from each patient in plain tubes (with no anticoagulants added). After clot formation, samples were centrifuged at 3000 × g for 10 min and serum was removed, aliquoted and stored at—20 ºC for estimation of serum level of vitamin D, total IgE, specific IgE for Dermatophagoides d1, d2 and specific IgE for other inhalant allergens. Laboratory investigations were done by ELISA TEST to evaluate level of total IgE by VIDAS Total IgE with instruments of the VIDAS family (VITEK Immuno Diagnostic Assay System) as an automated quantitative enzyme linked fluorescent immunoassay (ELFA) assay Kit made in France. It contained all the necessary reagents required for performing quantitative measurement of total IgE levels from samples. When the VIDAS Total IgE assay is completed, the results are analyzed automatically by the instrument, and a report is printed for each sample.

Serum 25 (OH) Vitamin D level was determined using VIDAS 25- Hydroxy Vitamin D (25-OH-D) kit with instruments of the VIDAS family (VITEK Immuno Diagnostic Assay System) as an automated quantitative enzyme linked fluorescent immunoassay (ELFA). The kite was made in France. It contained all the necessary reagents required for performing quantitative measurement of 25(OH) Vitamin D in samples. At the end of the assay, results were automatically calculated by the instrument in relation to the calibration curve stored in memory, and then were printed out.

### Specific IgE for inhalant allergens and dermatographoides

Enzyme immunoassay for semi qualitative detection of specific IgE antibodies for inhalant allergens and dermatographoides in human serum by ALLERQUANT with ELISA assay Kit made in China. It was stored at 2–8 °C. It contained all the necessary reagents. Stool samples were examined macroscopically and microscopically to exclude any patients with parasitic infestations.

### Ethical approval

This study was approved from Ethics Committee of Institutional Review Board (IRB) of the Faculty of Medicine, South Valley University prior to study execution. The study on humans were carried out in accordance with ethical guidelines and regulations. The trial was registered on the clinical trial registration website https://clinicaltrials.gov/NCT03847441. An informed consent was taken from all patients and from guardians of children. The choice of participants or withdrawal at any time without any obligation.

### Statistical analysis

Data were verified, coded by the researcher and analysed using IBM-SPSS 21.0 (IBM-SPSS Inc., Chicago, IL, USA). Descriptive statistics: Means, standard deviations, medians, ranges and percentages were calculated. Test of significances: chi-square test was used to compare the difference in distribution of frequencies among different groups. For continuous variables; independent t-test analysis was carried out to compare the means of dichotomous data, Man- Whitney U test was used to compare the median difference between groups that do not follow the normal distribution. Correlation analysis was used to test the association between variables (Spearman^'^s rank correlation. The clinical and demographic factors with proven statistical significance from the univariate analyses were further included in the multivariate logistic regression models. A significant p value was considered when it is equal or less than 0.05.

## Sample size calculation

Sample size calculation was carried out using G*Power 3 software^18^. A calculated minimum sample of 46 patients was needed to detect an effect size of 0.3 in the correlation between vit-D3 and anti-allergic antibodies (total IgE and specific IgE), with an error probability of 0.05 and 80% power on a two-tailed test.

## Results

The present study included 50 patients with AD with ages ranged from 2 months to 16 years old with a mean of 4.8 ± 2.7 years. 44% of patients were females while 56% were males. According to Rajka and Langeland (R & L) score and severity of atopic dermatitis among the studied sample, the mean R& L score was 5 ± 2 with a median of 4 (3–9). Regarding the severity of atopic dermatitis (as measured by R &L score), 54% (n = 27) had mild AD, 30% (n = 15) were categorized to have moderate AD and 16% (n = 8) had severe AD (Table 1).

**Table 1:** Severity of atopic dermatitis among the studied sample according to Rajka & Langel and Score.

Variable	Category	n = 50
R & L Score	Mean ± SD	5.00 ± 2.0
	Median (IQR)	4 (3–9)
Disease severity	Mild	27 (54%)
	Moderate	15 (30%)
	Severe	8 (16%)

The level of the total IgE among the AD patients ranged from 4 to 1300 UI/ml with a mean of 299 ± 70 UI/ml and a median of 192.

Regarding level of specific IgE for mites, 54% (n = 27) of samples had negative results and 44% (n = 22) had positive results (22% -n = 11- had d1 and 24% -n = 12 had d1 + d2). For the specific IgE for other inhalant allergens; 18 AD cases (36%) had negative test and 32 cases (n = 64) had positive findings (Table 2).

**Table 2:** Level of antiallergic antibodies of studied cohort.

Variable	Category	n = 50
Total IgE	Mean ± SD	298.60 ± 69.7
	Median (IQR)	192(4–393)
Specific IgE for Mites	Negative	27(54%)
	d1	11(22%)
	d1 + d2	12(24%)
Specific IgE for other inhalant allergans	Negative	18(36%)
	Positive	32(64%)

The mean level of vitamin D3 in the study group was 16.2 ± 11 ng/ml with a median of 9 ng/ml and a range of 8 – 90 ng/ml. Using 12 ng/ml as a cut-off for vit-D deficiency; about 60% of the sample (n = 29) had vitamin D deficiency and about 40% (n = 21) had a normal vitamin D level (Table 3).

**Table 3:** Level of vitamin D3 of the studied cohort.

Variable	Category	n = 50
Vitamin D3 level	Mean ± SD	16.19 ± 11.4
	Median (IQR)	9 (8–90)
Vitamin D3 deficiency	Yes (> 12 ng/ml)	29 (58%)
	No (≥ 12 ng/ml)	21(42%)

Comparative analysis between mild and moderate/severe AD regarding laboratory data showed that the total IgE median level was higher in cases with moderate/severe AD (192 UI/ml) as compared with (90 UI/ml) in mild AD which is statistically significant (p < 0.01).

Regarding specific IgE for mites; mild AD cases had statistically significant lower rates of positive results (14.8%) in comparison with moderate/severe AD cases (82.6%) (p < 0.001).

Likewise, mild AD cases had statistically significant lower rates of positive results of specific IgE for other inhalant allergens (48.1%) in comparison with moderate/severe AD cases (84.6%) (p < 0.05).

As regard level of vitamin D3, the median level among patients with mild AD was 19 ng/ml (8–90), while, among moderate/severe AD cases it was 8 ng/ml (7–12). There was statistically significant association between median vitamin D3 level and disease severity (p < 0.001).

Comparing vitamin D deficiency, all cases (100%) with moderate/severe AD had vitamin D deficiency as compared with only one-fifth of those with mild AD and this association was statistically significant (p > 0.001) (Table 4).

**Table 4:** Laboratory data differences according to disease severity.

Parameter	Mild (n = 27)	Moderate/severe (n = 23)	P value
Total IgE (median & range)	90 (5–393)	192 (4–1300)	= 0.009*
**Specific IgE for mites**			
Negative	23 (85.2%)	4 (17.4%)	< 0.001**
d1	2 (7.4%)	9 (39.1%)	
d1 + d2	2 (7.4%)	10 (43.5%)	
Level of vitamin D	19 (8–90)	8 (7–12)	< 0.001*
**Vit-D deficiency**			
Yes (< 12 ng/ml)	6 (22.2%)	23 (100%)	< 0.001**
No (≥ 12 ng/ml)	21 (77.8%)	0 (0%)	

*Independent Mann Whitney U test was used to compare the median differences between groups.

**Chi-square test was used to compare proportions between groups.

The univariate correlation between Vitamin D3 level and anti-allergic Antibodies in AD patients showed that there was a significant high moderate negative correlation with the total IgE level (r = − 0.55, p < 0.001). Likewise, there was a significant high negative correlation with the specific IgE for mites (r = − 0.62, p < 0.001) & vitamin D3.

On the other hand, there was non-significant minimal negative correlation with the specific IgE other inhalant allergens (r = − 0.10, p > 0.05) (Table 5).

**Table 5:** Correlation between Vit-D3 level and anti-allergic antibodies in patient.

Parameter	Vit-D3 Level (ng/ml)	
	r*	p value
Total IgE	− 0.546	< 0.001
Sp. IgE Mites	− 0.615	< 0.001
Sp. IgE other allergens	− 0.101	= 0.242

*Spearman’s rank correlation coefficient.

## Discussion

In spite extensive research, AD is still challenging disorder regarding its pathogenesis and management. There is a variation in prevalence, manifestations and severity of the disease worldwide according to many factors such as temperature, humidity, patient ages and other factors^13^. In the present study there was a variable age distribution ranging from infancy to adolescence and minor male predominance. Our findings support those of Lonny et al., 2015 who found that the incidence of eczema varies substantially with age and is highest in the first year of life, especially in boys^19^. In contrast, Silverberg and Hanifin, 2013 showed no sex predominance in early onset AD while in late onset AD it is more common in female^20^.

Results of the present study showed a significant high levels of non specific total IgE in patients with AD than normal levels. We also detected a significant higher level of total IgE in cases with moderate/severe AD as compared with those mild AD. These results concede with those of Yang et al., who found a positive correlation between serum IgE levels and AD severity^21^. In another study by Cheon et al., they found that the SCORAD index was positively correlated with total IgE levels^22^. Many other studies showed higher levels of IgE in AD patients and positively correlated with severity of the disease^23^. In contrast to the fore mentioned results, Ahmed & Nasreen^24^ disagree with our result and found that Total IgE may not be high in all patients with AD.

The present study found a statistically significant positive correlation between severity of AD & HDM specific IgE level with lower positive rates in mild AD in comparison with moderate/severe AD cases. Our results agree with those of Park et al., who found a positive relevance between both HDM sensitization and AD^25^. Many other studies detected positive correlation between specific HDM IgE and AD severity^26,27^. On other hand Yang et al., found that there was no significant relevance between both HDM sensitization and AD but they noticed elevation of HDM specific IgE in some patients with severe AD in their study^21^.

In this study, we found a statistically significant negative correlation between vitamin D level and disease severity. This finding is in agreement with findings of El Taieb and other colleagues who suggested that vitamin D deficiency may have a role in the development of AD and also they found a negative correlation between vitamin D and severity of AD^28^. Those results agree with findings of Ozlem et al., who demonstrated that vitamin D levels were significantly lower in moderate and severe AD compared with mild disease^29^. Some studies found no correlation between vitamin D level with either the development or severity of AD^21,30^.

Data from the present study suggest a correlation between vitamin D levels and severity of AD. We also found a negative correlation between vitamin D levels with both HDM specific IgE and total IgE. It was hypothesized that vitamin D deficiency has a role in immunologic dysregulation, impaired epidermal barrier function, and decreased cutaneous defense mechanism in patients with an AD^21^. It was found that decrease in vitamin D levels may increase the risk of HDM sensitization by enhancement of penetration of HDM through damaged skin barrier. Increased HDM sensitization may aggravate immunologic dysregulation which may increase the severity of AD^21^.

In conclusion, we found low vitamin D levels, high HDM specific IgE and Total IgE in patients with AD. Vitamin D was negatively correlated with severity of AD and both specific and total IgE. These findings suggest a relation between vitamin D deficiency and progression of AD through enhancement of HDM and other antigens sensitization. Further studies with large sample size and evaluation of other variables such as age, outdoor activity, seasonal variations, dietary habits and other atopic disease are required to support our data. These data advocate the need for larger studies to evaluate the use of vitamin D either by sun exposure, medications or dietary supplementation as a treatment option in patients with AD.

### Study limitations

Limitations of the present study included the small sample size and absence of a regression model as we could not adjust for seasonal variation, age and type of skin.
